# Supplementary material for: Individual-oocyte transcriptomic analysis shows that genotoxic chemotherapy depletes human primordial follicle reserve in vivo by triggering proapoptotic pathways without growth activation
Source: Sci Rep. 2021 Jan 11;11:407. doi: 10.1038/s41598-020-79643-x (PMC7801500; doi:10.1038/s41598-020-79643-x)
Supplement: Supplementary file 1 — Supplementary Information 1. [file 41598_2020_79643_MOESM1_ESM.docx]

**Title:** Individual-oocyte transcriptomic analysis shows that genotoxic chemotherapy depletes human primordial follicle reserve by triggering proapoptotic pathways without growth activation.

**Running Title:** Mechanisms of Chemotherapy-Induced Damage to Ovarian Reserve

**S. Titus^1^, K.J. Szymanska^1^, B. Musul^1^, V. Turan^1^, E. Taylan^1^, R. Garcia- Milian^2^,** **S. Mehta^3^, K. Oktay^1*^**

^1^ Yale University School of Medicine, Department of Obstetrics, Gynecology and Reproductive Sciences, New Haven, CT, USA

^2^ Bioinformatics Support Program, Yale School of Medicine, New Haven, CT, USA

^3^ Yale Center for Genome Analysis, Yale University, New Haven, CT, USA

***Corresponding author:** K. Oktay: correspondence@fertilitypreservation.org

**SUPPLEMENTARY DATA**

**Supplementary Video S1. Video showing laser capture microdissection (LCM) of a primordial follicle oocyte.** The slides are prepared from human ovarian tissue embedded in OCT medium. These slides were subsequently stained with hematoxylin and eosin to be able to identify follicle stages based on their morphology under the 40x magnification lens on Leica LMD7000 microscope. The oocyte is dissected from pregranulosa cells by using a laser-beam, allowing precision, and avoiding damage to the oocyte or incorporation of pregranulosa cells. We thank Justin Yazdi for the editing of the video clip.

**
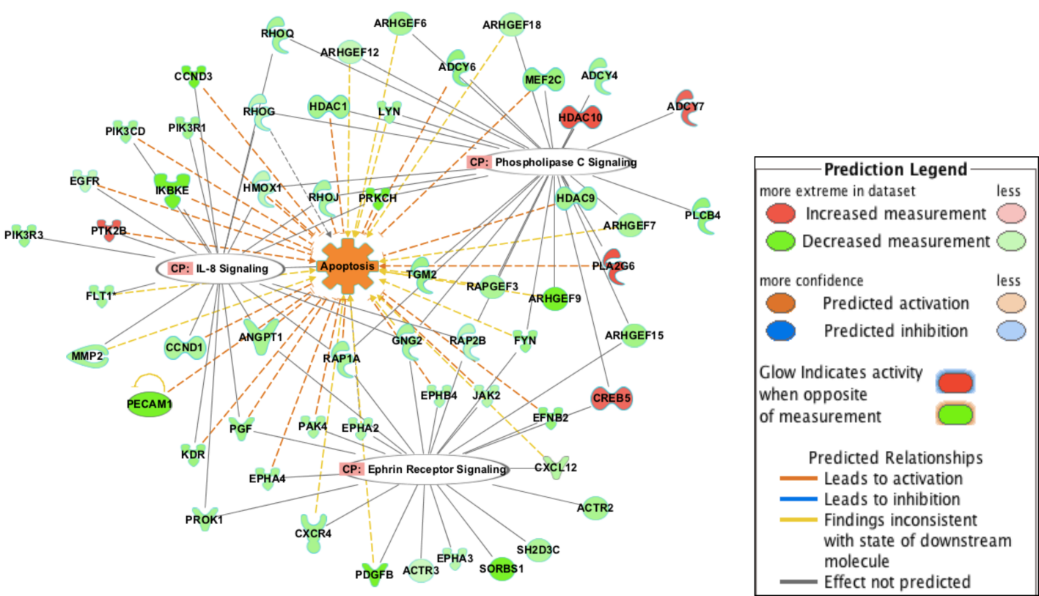
**

**Supplementary Figure S1. Detailed representation of altered genes in acute response to chemotherapy exposure in human primordial follicles.** IPA predicted activation of apoptotic processes in the cyclophosphamide-treated samples as there is a decrease in the expression of the genes regulating the phospholipase C, Ephrin and IL-8 signaling. A decrease in the expression of the genes of these anti-apoptotic pathways predicted activation of apoptosis in cyclophosphamides-treated primordial follicles.


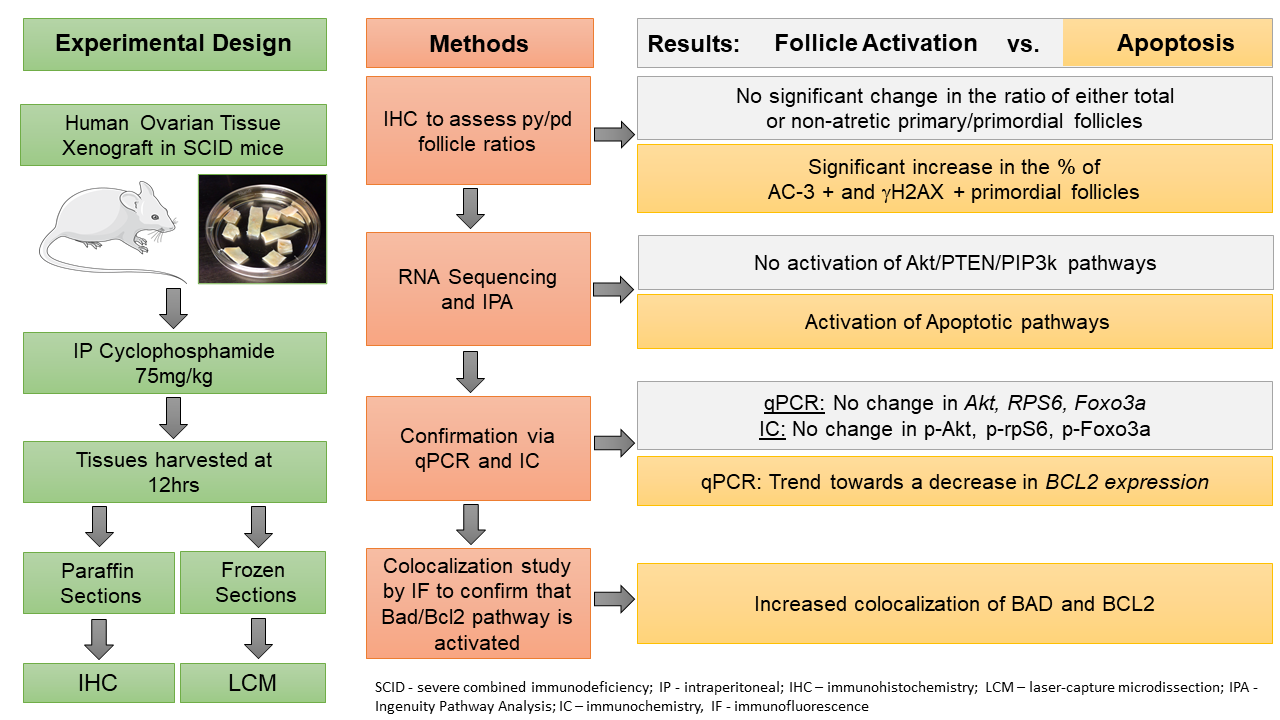
**Supplementary Figure S2. Summary of experimental design, methods and results.**

The figure used an image from Servier Medical Art.

**Supplementary Table S1. qRT-PCR primers for sequencing validation.**

| Gene | Forward Primer (5’ -3’) | Reverse primer (5’ - 3’) | Reference |
| --- | --- | --- | --- |
| βActin | GGACTTCGAGCAAGAGATGG | AGCACTGTGTTGGCGTACAG | [36] |
| Akt 1 | ATGAGCGACGTGGCTATTGTGAAG | GAGGCCGTCAGCCACAGTCTGGATG | [37] |
| rpS6 | CTGAACATCTCCTTCCCAGCCA | CCTTGTTTGTCGTTCCCACCAC | [38] |
| FOXO3a | AAATGAAAGCTCACTCTGGATTCC | TGTGCAATTCCTATGCAATC | [39] |
| BCL2 | GGTGGGGTCATGTGTGTGG | CGGTTCAGGTACTCAGTCATCC | [40] |
| BAD | CCCAGAGTTTGAGCCGAGTG | CCCATCCCTTCGTCGTCCT | [41] |

**Supplementary Table S2. Differentially expressed genes between the vehicle- and cyclophosphamide -treated groups.** Fold change ≥ 2, p<0.05.

| **Symbol** | **log2 Fold Change** | **P adj** |
| --- | --- | --- |
| **UBE3C** | -9.180882449 | 0.040085333 |
| **BTBD7** | -22.25567861 | 2.13E-09 |
| **PRKCH** | -20.51335026 | 3.31E-06 |
| **ARAP2** | -20.4954675 | 2.66E-06 |
| **MSMO1** | -16.0096814 | 0.008788797 |
| **PRDM1** | -10.20430463 | 0.043086696 |
| **MPPED2** | -19.48784036 | 9.01E-05 |
| **ING3** | 35.44168473 | 1.76E-11 |
| **RARB** | -17.30807194 | 0.000306996 |
| **RUNX1T1** | -22.03094937 | 1.74E-07 |
| **EXD2** | -16.9633179 | 0.000103482 |
| **MEF2C** | -10.16269657 | 0.045965997 |
| **COL19A1** | -18.3385071 | 0.001131939 |
| **SLCO1A2** | -19.96074333 | 0.000102151 |
| **ATRN** | -22.03906099 | 3.05E-09 |
| **SORBS1** | -23.7788134 | 2.03E-08 |
| **ERMP1** | -22.52222231 | 1.95E-07 |
| **PDGFB** | -21.26474926 | 1.01E-07 |
| **CACNA1I** | 29.19208883 | 4.34E-08 |
| **HDAC10** | 29.27917335 | 6.38E-08 |
| **SEC23B** | -23.23375918 | 4.17E-10 |
| **POLI** | -21.1819802 | 1.36E-08 |
| **DGKH** | -10.65301456 | 0.017627077 |
| **SLC30A4** | -19.60037782 | 0.000305686 |
| **DECR1** | -20.59045592 | 0.00011553 |
| **ARMC1** | -20.55842663 | 9.18E-08 |
| **CARD8** | -21.88104536 | 2.97E-09 |
| **MAG** | -17.7149294 | 0.001294348 |
| **MPP6** | -21.0782514 | 7.46E-07 |
| **SERPINE1** | 24.35415795 | 4.19E-06 |
| **SUSD1** | -20.4857159 | 0.000253897 |
| **MTMR4** | -22.0869566 | 7.31E-09 |
| **PNPO** | 33.6783901 | 1.61E-15 |
| **ENO3** | 18.94379419 | 0.000608386 |
| **AKAP10** | -10.44955145 | 0.028589419 |
| **CNTNAP1** | -23.15936896 | 2.88E-07 |
| **FRG1** | -18.03911976 | 0.000118365 |
| **CBL** | -9.640487536 | 0.046280656 |
| **VWF** | -12.99874622 | 0.010713752 |
| **PRDM4** | -23.32442267 | 1.91E-08 |
| **RIC8B** | -20.75051691 | 7.88E-06 |
| **CCND3** | -19.56491465 | 3.04E-06 |
| **LNPEP** | -21.02597365 | 1.09E-06 |
| **UMPS** | -16.24989683 | 0.000160938 |
| **TANC1** | -10.1182097 | 0.030314815 |
| **PDE1A** | -18.27341468 | 0.001166159 |
| **IL18R1** | 20.77140348 | 0.000102144 |
| **TIA1** | -10.24383351 | 0.008582286 |
| **NID1** | -9.92446493 | 0.040085333 |
| **UAP1** | 20.63352781 | 8.93E-06 |
| **PHF19** | -18.0354321 | 0.000937452 |
| **CLU** | -17.79400573 | 1.86E-05 |
| **RNF2** | -10.02982899 | 0.035465596 |
| **GTDC1** | -21.67052739 | 1.92E-06 |
| **ZC3H13** | -9.821040712 | 0.040657763 |
| **PFKFB2** | 17.68006707 | 0.001831132 |
| **FAM124B** | -26.63308197 | 1.79E-06 |
| **NCOA3** | -24.18374213 | 4.66E-11 |
| **CSE1L** | -10.13920406 | 0.049144593 |
| **EFNB2** | -10.78362224 | 0.040657763 |
| **CHCHD5** | -18.77963693 | 7.99E-06 |
| **CD93** | -10.81062304 | 0.048661808 |
| **CASD1** | -20.24726369 | 1.54E-06 |
| **ELL3** | -18.49467853 | 0.000213266 |
| **OLFM1** | 20.96703376 | 0.000108969 |
| **LAMA5** | -11.28066061 | 0.010797172 |
| **ARHGEF9** | -21.31265053 | 1.09E-08 |
| **NES** | -24.06359042 | 4.19E-09 |
| **PEMT** | 17.21280874 | 0.000366853 |
| **RFC3** | -15.64003897 | 0.003353584 |
| **ADAM30** | 15.06788772 | 0.030373776 |
| **RSAD2** | -18.80106 | 0.000116153 |
| **OSTF1** | -18.01732653 | 0.000134545 |
| **ADAM19** | -21.55762564 | 4.84E-07 |
| **ZC3H10** | -19.67488902 | 4.70E-05 |
| **MAP7** | -17.2512527 | 0.002866974 |
| **ATAT1** | -19.38991174 | 2.26E-06 |
| **SEMA6D** | -22.8897922 | 6.84E-08 |
| **SPTBN5** | -20.11399628 | 6.96E-06 |
| **GCOM1** | 20.07923462 | 2.34E-05 |
| **ENPEP** | -20.94283703 | 1.04E-05 |
| **N4BP2L1** | -17.88019831 | 4.54E-05 |
| **GREB1L** | -18.29158073 | 0.000669577 |
| **HSPG2** | -7.931575904 | 0.019330356 |
| **MOB3C** | -10.14987715 | 0.034658932 |
| **CTTNBP2NL** | -20.19057034 | 1.17E-06 |
| **RGS5** | -30.25289435 | 1.93E-09 |
| **HMCN1** | -9.689614575 | 0.042891939 |
| **GOLPH3L** | 20.46109531 | 1.33E-06 |
| **LYST** | -9.828521564 | 0.046280656 |
| **CEP170** | -21.78388943 | 2.77E-09 |
| **ATG12** | -9.792294539 | 0.042891939 |
| **ARHGAP26** | -20.32256738 | 6.66E-07 |
| **ZNF300** | -23.72328262 | 9.83E-11 |
| **MUT** | 20.18831251 | 1.93E-05 |
| **VWDE** | -22.09349921 | 4.71E-08 |
| **NRBF2** | 21.13521377 | 5.76E-06 |
| **IGSF10** | -17.58703584 | 0.000411629 |
| **SPARCL1** | -22.23919408 | 5.51E-08 |
| **MR1** | 18.49995251 | 0.00011994 |
| **RGPD3** | -16.08061108 | 0.007400237 |
| **AFAP1L1** | -30.17985081 | 4.17E-10 |
| **MPZL3** | -15.9565452 | 0.006328824 |
| **BBS5** | 17.00645382 | 0.002974405 |
| **ATP1A1** | -10.02617289 | 0.034931684 |
| **EMCN** | -30.89778162 | 3.09E-10 |
| **AL353743.1** | -18.6023219 | 0.000359661 |
| **ARMC3** | -16.67429762 | 0.0031464 |
| **C10orf10** | -23.44177534 | 2.92E-10 |
| **ZNF143** | -20.35430683 | 2.20E-06 |
| **MCM7** | -23.85132366 | 9.80E-09 |
| **TERF2IP** | -21.134117 | 3.40E-07 |
| **TRANK1** | -24.35123465 | 2.02E-10 |
| **REEP4** | 34.4432497 | 3.81E-11 |
| **TSPAN5** | -21.2573024 | 4.18E-07 |
| **SLC35G2** | -30.39454241 | 1.36E-10 |
| **LDB2** | -30.66872459 | 1.33E-08 |
| **MAP3K2** | -10.01730122 | 0.047922213 |
| **COMMD5** | -21.8663568 | 2.67E-06 |
| **ZNF16** | -21.07545007 | 1.62E-07 |
| **KBTBD2** | -10.26573269 | 0.033774936 |
| **KCND3** | -17.8810868 | 0.001166159 |
| **SLC19A1** | -12.28347574 | 0.001308306 |
| **ADCY6** | -10.55009928 | 0.010073599 |
| **CMKLR1** | -17.78361551 | 0.000289413 |
| **PAAF1** | -21.34019035 | 4.84E-07 |
| **CCDC57** | -19.4782915 | 2.52E-07 |
| **SGF29** | 21.19938757 | 9.56E-09 |
| **RIMKLA** | -21.56671249 | 3.07E-06 |
| **GPC5** | -15.77015927 | 0.010599268 |
| **DNHD1** | -21.12993266 | 2.36E-08 |
| **ANKRD18A** | 30.72388602 | 8.59E-09 |
| **ZDHHC20** | -22.05966297 | 2.24E-06 |
| **RNF135** | -16.92388324 | 0.004277318 |
| **DEXI** | -17.56646109 | 0.001831132 |
| **ADGRG3** | 16.7874542 | 0.002302511 |
| **ZFP1** | -20.52784498 | 5.34E-06 |
| **KIAA0825** | -18.93213716 | 0.000305518 |
| **GPRIN3** | -19.13502957 | 1.24E-05 |
| **PRKG1** | -32.83887845 | 1.29E-10 |
| **LAMP1** | -21.89685688 | 2.40E-09 |
| **ANKRD37** | 17.68434945 | 5.96E-05 |
| **TNFRSF4** | -19.81579227 | 0.000389422 |
| **ZNF559** | -9.720558653 | 0.046280656 |
| **NDOR1** | -17.74098876 | 0.000181783 |
| **ZNF548** | -18.70994871 | 1.15E-06 |
| **S100A4** | -21.15527224 | 0.0002424 |
| **DACT3** | -18.60073437 | 0.000253897 |
| **DCHS2** | 22.13441408 | 3.93E-06 |
| **ENTPD7** | -21.02901966 | 9.22E-07 |
| **FAM19A2** | -16.36225354 | 0.003249114 |
| **COL15A1** | -11.79653071 | 0.016588591 |
| **ABHD16A** | 21.2288493 | 0.000141662 |
| **GCNT6** | 26.26270332 | 2.55E-06 |
| **CRYZL1** | -10.19429058 | 0.031644741 |
| **SEC14L1P1** | 17.59586167 | 0.000366705 |
| **PLEKHM1P1** | -19.7226533 | 2.26E-06 |
| **LINC00680** | -19.75272435 | 0.000123171 |
| **RPL17-C18orf32** | -18.07827757 | 0.003007074 |
| **AC068491.1** | -29.63228622 | 3.54E-08 |
| **OR52I2** | 29.73227328 | 9.17E-09 |
| **AC092641.1** | 29.88828572 | 2.08E-08 |
| **SATB1-AS1** | -19.50370225 | 3.24E-05 |
| **SNORA71B** | -16.83514163 | 0.007730674 |
| **AL035448.1** | -21.82599564 | 3.30E-06 |
| **MIR600HG** | -19.91330603 | 2.41E-05 |
| **PRMT5-AS1** | -17.38168289 | 0.000267746 |
| **AP001992.1** | -18.65959609 | 0.000102723 |
| **ACTG1P13** | 29.25362388 | 2.27E-08 |
| **MCPH1-AS1** | 32.28278911 | 5.39E-11 |
| **AC026904.3** | -26.13288878 | 2.81E-06 |
| **AC091053.1** | -21.32092916 | 4.03E-07 |
| **CTSO** | -18.84393824 | 9.01E-05 |
| **TAS2R31** | -15.71595565 | 0.015553005 |
| **TMC3-AS1** | -16.03332764 | 0.003047062 |
| **TYRO3P** | 18.18361836 | 0.000461693 |
| **AC009093.1** | 10.27179944 | 0.047922213 |
| **AC127459.1** | -20.0885839 | 1.09E-06 |
| **PECAM1** | -25.19837655 | 2.13E-09 |
| **PAN3-AS1** | -17.41081947 | 0.001976919 |
| **IKBKE** | -16.9791079 | 0.001656132 |
| **AP000654.1** | 26.42316847 | 1.55E-06 |
| **ZNF2** | -34.49614293 | 5.32E-11 |
| **PADI6** | 25.65424808 | 4.55E-06 |
| **HIST1H4E** | -19.52678387 | 1.12E-05 |
| **BACE1-AS** | -16.08386962 | 0.000768391 |
| **AP000866.6** | -21.67792921 | 2.85E-05 |
| **AC008536.3** | -21.52395032 | 2.67E-06 |
| **AL590434.1** | -20.66578411 | 0.000119772 |
